# New insights into the origin of remote PPG signals in visible light and infrared

**DOI:** 10.1038/s41598-018-26068-2

**Published:** 2018-05-31

**Authors:** Andreia V. Moço, Sander Stuijk, Gerard de Haan

**Affiliations:** 1 Department of Electrical Engineering, Eindhoven University of Technology, 5612AZ Eindhoven, The Netherlands; 2 Philips Innovation Group, Philips Research, High Tech Campus 36, 5656AE Eindhoven, The Netherlands

## Abstract

Remote photoplethysmography (PPG) is an optical measurement technique with established applications in vital signs monitoring. Recently, the consensual understanding of blood volume variations (BVVs) as the origin of PPG signals was challenged, raising validity concerns about the remote SpO_2_ methodology. Recognizing the imperative for new opto-physiological evidence, this investigation supports the volumetric hypothesis with living skin experiments and Monte Carlo simulations of remote PPG-amplitude in visible light (VIS) and infrared (IR). Multilayered models of the skin were developed to simulate the separate contributions from skin layers containing pulsatile arterioles to the PPG signal in the 450–1000 nm range. The simulated spectra were qualitatively compared with observations of the resting and compressed finger pad, and complemented with videocapillaroscopy. Our results indicate that remote PPG systems indeed probe arterial blood. Green wavelengths probe dermal arterioles while red-IR wavelengths also reach subcutaneous BVVs. Owing to stable penetration depths, the red-IR diagnostic window promotes the invariance of SpO_2_ measurements to skin non-homogeneities.

## Introduction

**Figure 1:**
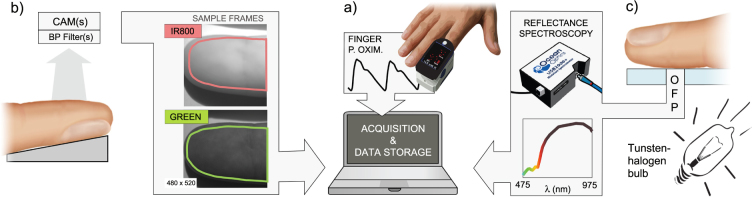
PPG signals can be acquired (**a**) in transmission-mode, e.g., by finger pulse oximetry, (**b**) remotely, or (**c**) in reflectance-mode, e.g., by using a spectrometer (OFP, optical fiber probe). The figure was created by A. Moço.

Photoplethysmography (PPG) is an optical measurement technique that has advanced technically, achieving ubiquity in current clinical settings^1^ and the status of enabling technology for non-obtrusive innovations in pulse-rate and SpO_2_ monitoring^2–5^. Various sensing modalities are available for probing PPG signals. Its fundamental distinction is in whether signals are acquired in transmission or in reflection mode. Transmission-based acquisition requires the illuminating source and photosensor to face opposing sides of the tissue, whereas the latter has these elements on the same side. Transmission-mode PPG results from minute cardiac-related modulations of skin absorbance and is ubiquitous in most finger pulse oximeters (see Fig. 1a).

Camera-based systems can operate in transmission-mode^6^, but are better suited for remote reflectance measurements. Remote PPG allows that the pulse-rate is extracted, preferentially, at green wavelengths, though a quest for motion robustness deems necessary that multiple wavelength bands (color-channels) are combined^7^. The red-IR diagnostic window has also been shown suitable for PPG-based measurements, including SpO_2_^8^.

PPG systems continue to mature by exploring of the PPG signal’s frequency diversity. Multispectral PPG data, in VIS–IR, may find clinical value in skin health assessments^9,10^. While multispectral cameras remain prohibitively expensive and computationally heavy, multispectral PPG can be acquired by coupling a spectrometer with an optical fiber probe (OFP; see Fig. 1c). Since there is contact of the probe with the skin, we refer to this setting as reflection-mode acquisition.

## Importance of investigating the origin of the PPG signal

It may come as a surprise that the PPG-based techniques and applications have developed more than the opto-physiological knowledge pertaining to the origin of the signal, which remains vaguely referred to as arterial blood volume variations (BVVs) occurring at every cardiac heart beating within the microvascular bed of tissue. Still, the understanding of PPG as BVVs, which we shall refer to as the volumetric model, has been able to support the working principle of current PPG-based applications. In particular, the volumetric model provides a rationale for using PPG as a surrogate of the arterial blood oxygenation curve. The enabling principle is the dependency between normalized PPG-amplitude ratios at red–IR wavelengths, and the relative proportion between oxygenated and non-oxygenated haemoglobin absorption^3,11^.

The volumetric model is consensual but not unique^1^. For example, flow variations have been suggested as a mechanism for inducing light reflectance variations^12,13^. Naslund *et al*.^12^ observed reflection-mode PPG signals by using IR wavelengths in the patellar bone, a site where vessel wall distentions are not theoretically possible. It remains uncertain whether this observation is spurious contamination by surrounding pulsatile tissue. More reliable optical insights on PPG generation may be obtained by investigating blood flow in phantoms. Lindberg^13^ showed that blood reflection changes if a liquid solution containing red blood cells (RBCs) flows in a rigid tube. When the flow velocity and concentration of the solution are such that periodic RBC aggregation takes place (i.e., haematocrit levels above 38%), the blood reflection reflects the changes in the orientation and deformability of the RBCs^13^. On a similar setup, Shvartsman^14^ simulated pulsatile blood flow and confirmed that PPG-like signals are associated with geometric changes in RBC aggregation^14^.

Recently, Kamshilin *et al*.^15^ reported conflicting observations with the volumetric model^16^. One of such observations were counter-phase PPG signals in the vicinity of the radial or brachial artery, which are simply motion artifacts^17,18^. A more interesting argument raised against the volumetric model was the apparent paradox that the PPG-amplitude peaks in green, although it would not, theoretically, even reach pulsating arterioles. Gently compressing the skin against a glass plate increases the green PPG-amplitude further, which, again, appears to find no explanation in the volumetric model.

Consequently, a new theory was proposed, drawing attention to elastic deformations of the dermis as dominant mechanism of PPG formation. The increasing transmural pressure of the arteries during systole would compress the dermal connective tissue and increase the overall the capillary density. These deformations would explain the observed gains under compression. However, if arterial BVVs are not the origin of PPG, concerns emerge when it comes to the validity of PPG-based SpO_2_ measurements. In fact, if the total dermal tissue is periodically compressed, then PPG-based SpO_2_ readings would not be possible as these would be severely contaminated by venous pulsations.

Supporting and broadening the application scopes of PPG requires that the depth-origin of the signal is confirmed and its origin explained. Thus, it is imperative that the volumetric model is revised in light of the recent experiments devised by Kamshilin^15^. If it could be shown that even visible light penetrates deep enough to interact with arterioles, then the confidence on the volumetric model would be enforced. In this paper we tackle this topic under the hypothesis that the volumetric model is true and take a combined numerical and experimental approach. The skin was modeled as multilayered media with optical properties that translate its anatomophysiology^19^. Then the Monte Carlo method was applied to simulate the PPG-amplitude spectra; its verification on living skin validated, indirectly, the volumetric model assumption.

## Modeling the skin’s microvasculature

**Figure 2:**
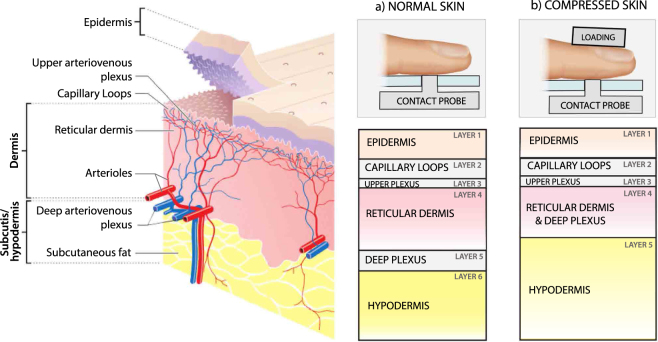
Skin represented as multiple stacked horizontal layers for (**a**) normal conditions and (**b**) for compression. The normal skin is modeled as epidermis (EPI); capillary loops (CL); upper plexus (UP); reticular dermis (RD); deep plexus (DP); and subcutis (SC). The compressed skin has reduced blood content and allows the light to penetrate deeper into the skin. On a contact-based setup, compression is achieved by loading the finger pad against the probe. The figure was built by A. Moço upon the skin layers of Madhero88, via Wikimedia Commons (en.wikipedia.org/wiki/File:Skin_layers.png, CC BY-SA 3.0).

The inhomogeneity of the microvasculature was accounted by representing the non-glabrous skin structure as a medium split into six stacked horizontal layers (see Fig. 2a). The first layer corresponds to the epidermis (EPI), which is bloodless^20^ and consists of mostly dead or dehydrated cells and no melanosoms. The thickness of the epidermis is highly skin-site dependent, but 0.8 mm is reasonable for the finger pad. The dermis was subdivided into four layers with different blood concentrations, which are as follows^21^: capillary loops (CL; 150–200 *μ*m thick); upper plexus (UP; 80 *μ*m thick), reticular dermis (RD; 1400–3000 *μ*m thick); and deep plexus (DP; 80–700 *μ*m thick). The arterial compartment in the plexuses and RD represent arterioles supplying the entire tissue volume and venules collecting the returning venous blood. The deepest layer of the model is the subcutis (SC; or hypodermis), which accounts for fat, connective tissue and pulsating arterioles or arteries.

Our hypothesis that BVVs are located at the dermal plexuses, RD and SC is partly in agreement with prior work of Reuss^22^, where it is assumed that only the plexuses contribute to PPG formation, but not with Huelsbusch^23^, who modeled the PPG’s origin at the capillary loops only. However, Huelsbusch’s PPG-amplitude spectrum largely underestimated the relative amplitude of signals in red-IR wavelengths, suggesting the incorrectness of this parameter setting.

We implemented models that mimic the opto-physiological contribution of the skin layers to the total reflectance-mode PPG spectra, separately, for normal and compressed skin (see Fig. 2a,b, respectively). Recognizing each of these contributions as the layer’s “signatures”, the overall remote PPG signal is in fact a mixture of the dermal and subdermal signatures. A useful as proof-of-concept for the volumetric model hypothesis is to isolate and demonstrate these signatures. However, the mixing weights are unknown, which is also why the compression intervention is valuable in this study.

Strong compression, yet below the systolic pressure level, is a simple intervention to block the dermal BVVs and blanch the skin, hence isolating the signatures from deeper pulsating structures in the PPG spectra of the compressed skin and allowing the incident light to reach deeper layers. Under full occlusion of the upper dermis, only the DP and SC remain pulsatile, and the removal of venous blood leads to its contributions being much stronger than in normal conditions. When sustained, metabolites accumulate and arterial vasodilation is also triggered^24,25^. To translate these effects in simulations, the compressed skin is modeled by the similar layers as the normal/reference skin (1–EPI; 2–CL; 3–UP; 4–RD & DP and 5–SC & digital artery). However, reductions are made to blood and water concentrations, layer’s thickness, whereas the relative strength BVVs from deeper layers is boosted. We remark that both skin models translate biological tissue heterogeneity in the depth dimension only, which is suitable for pointwise diffuse reflectance spectroscopy (DRS) measurements.

The skin layers signatures were generated by perturbing the diffuse reflectance of the skin’s model. Specifically, a systolic event corresponds to an influx of arterial blood to the skin tissue and was implemented as minute increments in the absorption coefficient of the dermal and subdermal layers with respect to diastolic-state reflectance of the model. Whenever possible, parameters conform to the literature. Otherwise, these were calibrated based on our own camera-based/remote measurements.

The next section is devoted to the results and discussion of our measurements and simulations. A methods section follows with a detailed description of the videocapillaroscopy protocol and implementation details of the Monte Carlo simulations.

## Results and Discussions

### Experimental Setups

This study comprises two experimental parts. Part 1 includes a comparison of PPG signals acquired with cameras in the green wavelength range and in IR, as well as insights from videocapillaroscopy and DRS. This part offers helpful observations for calibrating skin tissue simulations. For convenience, skin measurements were performed at the human finger (pad and nail fold). The skin of the finger pad is glabrous (non-hairy) and offers the advantages of strong PPG signals, negligible melanin content and ease of stabilization. The nail fold also allows acquisition of strong PPG signals and has the peculiarity of having the capillary loops oriented in parallel to the skin surface, which enables their observation by regular reflectance microscopy. Part 2 compares simulations and measurements of the PPG spectra in normal and compressed skin.

### Experimental Part 1: Multimodal remote PPG observations

#### Camera-based PPG in VIS versus IR

**Figure 3:**
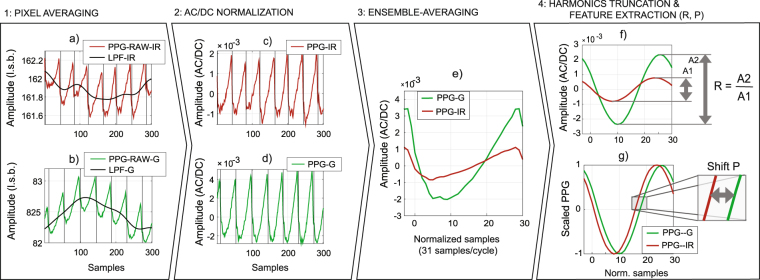
Processing pipeline: (1, 2) Remote-PPG signals acquired at the finger pad are low-pass-filtered and AC/DC-normalized; (3, 4) The AC/DC streams are ensemble-averaged (EA) (template size, 31 samples per cardiac cycle), including (**e**) multiple of the pulse-rate frequency and (**f**,**g**) the fundamental only.

Figure 3 illustrates our framework for comparing PPG signals in VIS-green and IR with data acquired on a subject from this study (SpO_2_ level, 95–98%; seated position) for a finger pad measurement performed 20–30 cm below the heart level (imaging area, about 100 mm^2^). The pad was measured with a monochrome camera (sampling rate, 30 Hz) under stable lighting conditions (incandescent bulb; 9 V, DC). Two consecutive measurements were performed using optical bandpass filters that isolate the green wavelength range (center frequency/unilateral bandwidth, 559/34 nm) and in IR (800/12 nm). Specifically, Fig. 3a,b show the average skin pixel intensity in consecutive samples for the green and IR recordings; i.e., “raw” PPG signals are the absolute diffuse reflectance of the skin over time. Similarly, Fig. 3c,d illustrate the normalized signals, resulting in periodic and zero-mean temporal series whose amplitude is typically upper bounded by 0.1. Consistent with the literature^2^, we denote this format as “AC/DC” (abbreviation,“alternate current” over “direct current”). AC/DC normalization is performed by dividing the “raw PPG” (units, least significant bits; l.s.b.) by its low-pass filtered component (LPF).

After pixel averaging and normalization, two periodic and light-invariant PPG streams are obtained. These are polluted by sensor noise but ensemble-averaging (EA) cardiac cycles eliminates noise while retaining signal information (see Fig. 3e). Note that the amplitude difference between green and IR signals is consistent with the wavelength dependence of PPG and with the expectation that green and IR interact with the vasculature at different depths^9^.

Waveform dissimilarity is further evidenced when the EA waveforms are truncated to the fundamental of the pulse-rate frequency and scaled to unity (see Fig. 3f,g). An obvious feature for quantifying dissimilarity between two waveforms is the relative amplitude w.r.t. a reference wavelength. For example, when the reference is set at 800 nm, a ratio-of-ratios, *R*, can be computed as the standard deviation of the normalized green over IR (center wavelength, 800 nm) waveforms. For the pair of recordings depicted in Fig. 3, we measured an R of 2.9, but inter-individual differences can be large. On a small sample size (*N* = 4), R was estimated to be 1.8 ± 0.8. Subsequently, this range will be considered for calibrating the remote PPG spectra. Another useful feature for quantifying dissimilarity is the phase shift, *P*, which we illustrate at the fundamental of the signals. A non-zero *P* between wavelengths supports that the microvasculature is probed at different depths. In Fig. 3g, P was measured as 20 degrees, but test-retest experiments in other subjects suggest that the range of *P* is broad, ranging up to 30 degrees.

#### Videocapillaroscopy

**Figure 4:**
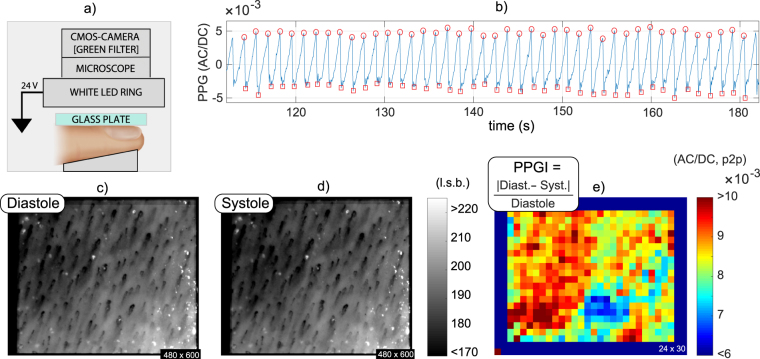
Videocapillaroscopic examination of the finger nail fold using green wavelengths: (**a**) Setup schematics; (**b**)Average PPG signal, with indication for systolic maxima (circles) and minima (squares); and super-resolved images of the upper dermis at the (**c**) peak instants and (**d**) valleys, respectively (imaging area, about 1.6 × 2 mm; amplification, ×60). The downsized and normalized differential image between (**c**,**d**) is a PPG-image (**e**) which suggests no relation between capillary density and PPG-signal strength. The figure was created by A. Moço.

Observing the capillary loops at the finger nail fold during PPG signal acquisition is insightful to investigate a possible contribution of capillaries to PPG. Owing to the low epidermal thickness at the nail fold, the capillary loops and arterioles are found as close to the surface as 0.28–0.43 mm and >0.43 mm, respectively^26^, and can be reached using green light. Figure 4a shows our videocapillaroscopic setup and Fig. 4b a stable PPG segment, overlapped and its peaks and valleys in one subject.

When the frames corresponding to these critical instants are registered and averaged, separately for systole and diastole, the corresponding super-resolved images of the capillary loops are obtained (see Fig. 4(c,d)). The peak-to-peak (p2p) amplitude of the signal remains fairly stable during the selected segment and no differences were apparent in the density of visible capillaries. An useful approach to continue exploring data is to perform the normalized difference between the systolic and diastolic frames. The outcome is a PPG-amplitude image (PPGI, amplitude expressed as AC/DC-p2p; see Fig. 4e) which indicates that the PPG-amplitude is strongest where the blood concentration is highest (identified as darker regions in Fig. 4d). This interpretation is not confounded by the local density of capillary loops, which is fairly even across the imaged area. PPGI further shows that the gradient of the PPG strength varies smoothly across the skin surface and does not reflect the activity of isolated or clustered loops. This supports the hypothesis that the PPG in green is modulated by upper dermal arterioles.

#### Spectroscopic measurements of PPG on normal and compressed skin

**Figure 5:**
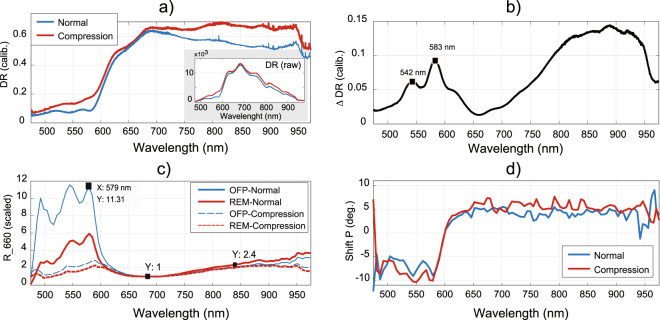
(**a**) DR profiles measured before and under skin compression; (**b**) the DR difference evidences the oxyhemoglobin absorption peaks. The cancellation of PPG at the VIS (<580 nm) range by compression is seen at the (**c**) scaled PPG-amplitude measurements (R_660; OFP, optical fiber probe; REM, remote PPG). The (**d**) PPG-phase spectra shift at about 600 nm, which indicates shape dissimilarity between VIS and IR.

This section shows our diffuse reflectance (DR) and reflectance-PPG spectra. 16 subjects were measured at normal conditions and under compression. One subject was excluded because the PPG signal in normal conditions was hidden by sensor noise. Figure 5a contrasts average DR spectra from the remaining 15 subjects of our dataset. The difference between these plots, ΔDR, is a wavelength-dependent function with relative peaks close to haemoglobin absorption (542 nm, 582 nm; see Fig. 5b).

Figure 5c shows the medians of the PPG spectra obtained with the photometer-OFP system at normal and compressed skin (N = 15). Also included are the corresponding remote spectra estimated by using a transfer function (TF), which accounts for the skin properties and probe specifications (see the methods section for details). To ease comparisons, the PPG-amplitude spectra are scaled by its relative minimum at 660 nm and two variables were defined: the amplitude ratio of green over red (GoR, with green and red ranges defined at 520–577 nm and 660–700 nm, respectively), and the ratio of IR (range, 800–840 nm) over red (IRoR). In our dataset, paired-sample t-tests indicated that compression significantly reduces the average GoR by a factor of 4 (means ± standard deviations; reference, 927 ± 319; compression, 2.32 ± 1.24; 1 rejected outlier; p < 0.001), whereas IRoR is not significantly affected (reference, 1.90 ± 0.16; compression, 1.95 ± 0.14; p = 0.43). This supports the multilayered BVVs hypothesis by evidencing that selectively blocking dermal layers affects the shallow PPG-green wavelengths. Note that the invariance of IRoR to compression holds for remote acquisition because the probe geometry is irrelevant in the red-IR diagnostic window. That green and red-IR wavelengths probe different mixture-weights of the layers signatures explains the selective mitigation of the PPG-amplitude below 580 nm by compression.

Figure 5d contrasts the relative PPG-phase shift spectra in the 475–975 nm wavelength range. The dual-state behaviour of the relative PPG-phase function, with stable phases within the blue-green (475–580 nm) and red-IR (625–975 nm), means that the shapes of the PPG signals are fairly similar within the ranges. The phase gap at ~600 nm indicates the abrupt penetration depth change, which is influenced slightly by the compression-induced blanching of the skin.

### Experimental Part 2: Monte Carlo simulations

**Figure 6:**
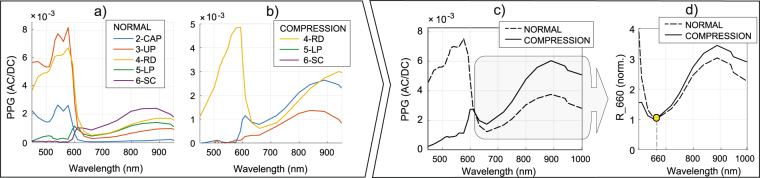
Skin layers signatures simulated for (**a**) reference and for (**b**) compression conditions, evidencing that deeper layers contribute the least to PPG in blue-green. Upon linear combination with weights *w*_*ref*_ and *w*_*comp*_, the remote PPG spectra are provided as (**c**) AC/DC and (**d**) relative to the amplitude at 660 nm.

The simulated layer’s signatures for normal and compressed skin are depicted in Fig. 6(a,b). The simulated remote PPG spectra resemble measurements for, e.g., pulsation patterns of *w*_*ref*_ = (0, 0, 1, 2, 3, 1)/3 and *w*_*comp*_ = (0, 0, 0, 2, 18, 1)/3, for layers (1-EPI, …, 6-SC), for reference and compression, respectively.

**Figure 7:**
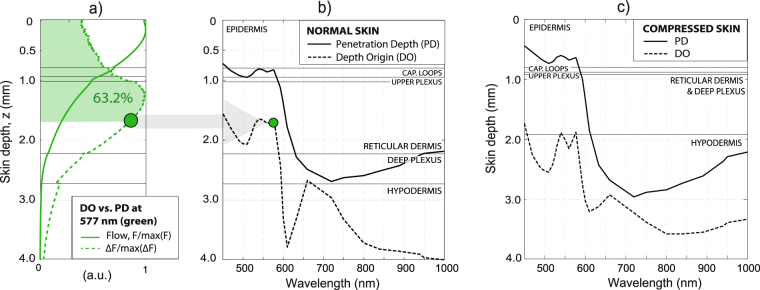
Comparison of the penetration depth (PD) of the incident light at the skin against the depth-origin (DO) of PPG for (**a**,**b**) normal and (**c**) compressed skin, showing that PPG modulations reach pulsating layers and have arteriolar origin in VIS-IR. As exemplified for 577 nm, the PD is determined as 63.2% of the area under the diastolic or systolic flow curves since *F*^(*d*)^ ≈ *F*^(*s*)^. Analogously, the DO of PPG is calculated from the diastolic-systolic difference Δ*F* = |*F*^(*d*)^ − *F*^(*s*)^|.

The simulations for the penetration depth (PD) and depth-origin (DO) of the PPG signals are insightful to assess if arteriolar BVVs can, at least theoretically, modulate PPG. Figure 7a exemplifies the light flux, *F*(*z*), and differential flux between diastole and systole, Δ*F*(*z*), for 577 nm. The PD is defined as the depth for which area under *F*(*z*) is ~63.2%. Analogously, the DO is defined as the depth for which the area under Δ*F*(*z*) is ~63.2%.

In agreement with earlier work^15,23^, we verified that the PD of blue-green wavelengths is at the level of the capillary loops (see Fig. 7b). However, only a small fraction of the diffusely reflected photons need to be modulated for PPG signal generation. The DO for the reference skin model is greater than the PD. This means that PPG in VIS effectively reaches the pulsating arterioles of the upper plexus and the RD. These insights also hold in IR; i.e., the center of gravity of the DO of the PPG signals in VIS-IR is deeper than the PD of the incident light. Similar insights hold for the compressed skin model (see Fig. 7c).

## Discussion

This investigation aims to assess if the opto-physiology of remote PPG in VIS-IR can be explained by arteriolar/arterial BVVs located at dermal and subdermal skin layers. Using the finger pad as inspection site, we showed that the remote PPG-amplitude spectrum for normal and for compressed skin can be acquired and modeled in light of the volumetric model. Skin compression reduces the blood content of the skin (particularly venous blood) and enables that green wavelengths penetrate deeper and reach more pulsating vessels. Since the depth-origin of the PPG signals is within the arteriolar level in VIS and IR, an alternative model for the genesis of PPG in VIS is, therefore, unnecessary. Yet, we remark that our insights are not direct experimental evidence and do not invalidate the possibility of complementary mechanisms of PPG formation occuring in parallel.

The volumetric model holds for all skin sites but the PPG spectra is have skin-site variations. Our preliminary results suggest lower overall PPG-amplitudes and an imbalance between amplitudes in green versus red-IR wavelengths, for glabrous and non-glabrous skin (see Section 2 in the Supplementary file). Possible explanations include density of arterio-venous shunt density, microvascular bed thickness and epidermal scattering and absorption.

To the best of our knowledge, only Reuss^22^ stated explicitly that the capillary loops are microcirculatory, but there is no experimental support for this assumption. At most, capillary flow velocities can be estimated and shown to have a constant and a pulsatile component. Based on nailfold videocapillaroscopy, the typical RBC average velocity measured in healthy subjects is around 0.8 ± 0.2 mm/s^27^. More recently, Baran *et al*.^28^ applied Doppler optical microangiography (DOMAG) method to map RBC absolute velocity in the arterial and side of the finger cuticle capillaries loops and obtained about 0.67 mm/s for arteriole-end capillaries. Unfortunately, the sampling rate was limited to 0.5 frames per minute, which did not allow the assessment of pulsating flow inside the capillary loops. Still, the fact that RBC speed even reduces in capillary loops^28^ with increasing flow resistance at low velocities^29^ suggests the steadiness of blood flow at upper arterioles. The assumption of constant flow velocity at the capillaries is not subscribed by Huelsbusch^23^, who modeled PPG assuming that the PPG is formed at the capillary loops only. Interestingly, Huelsbusch’s simulated spectrum largely overestimates the magnitude of signals in blue-green wavelengths, suggesting the incorrectness of this parameter setting. We verified that the problem does not occur in simulations where the sources of pulsatility/BVVs are at the dermal plexuses, RD and SC.

Recently, Volkov *et al*.^30^ showed that the capillary flow speed of RBCs of the fingernail fold have a pronounced pulsatile flow component overlapped with an asynchronous component. The magnitude of the capillary flow speeds (range, 1–5 mm/s) is dissonant from reference healthy ranges, but the observed morphological resemblance between capillary flow speed waveforms and pulsatile blood pressure waveforms is invariant to possible scaling inaccuracies. Still, these waveforms were erroneously interpreted as evidence for the inadequacy of volumetric theory. In fact, if the data of Volkov *et al*. is valid, then any remnants of pulsatile pressure that reach the capillary level are accommodated as pulsatile flow and not as capillary BVVs. This possibility is strengthened by morphological resemblance between Volkov’s flow speed waveforms and the pulsatile pressure waveforms of Mahler *et al*.^31^, who performed direct cannulation of human finger nailfold capillaries.

### Limitations and future work

#### Modeling simplifications

We begin by acknowledging the simplifying assumption of a reference skin model and PPG spectrum. This exercise should only hold for illustrative purposes because PPG is highly influenced by individual and contextual factors like posture^32^ and skin site (see Section 2 in the Supplementary file for details). Additional skin models with slight changes in the layers properties, e.g., thickness, relative pulsatile strength and absorption, would add to this study by translating inter-individual and skin-site variations. Still, building and running these come at the cost of added computational effort and time while a single skin model suffices to verify that the normal and compressed PPG-amplitude spectra are obtained for realistic parameter settings.

We further remark that modeling the skin as a structure with a discrete number of stacked horizontal layers is a valid simplification^33^, though not without the possible risk that the microanatomic description of the skin into 2 plexuses may not be representative of all skin sites^34,35^. Wong and Geyer visualized the finger pad using optical coherence tomography (OCT) and documented a tree-like ramification where relatively thick dermal arteries arise from the subcutaneous arterial plexus and ramify until they form the ascending segment of the capillary loops. As reported, capillary loops of the finger pad could be split in “arterial units” and the upper plexus would be absent. However, the volumetric model for PPG signal formation also holds if the skin microvasculature has a tree-like arrangement, although the mixing weights of the layer contributions to the resulting PPG spectrum may differ at the upper dermis. Future work is valuable to ascertain these considerations.

#### Parameter errors

Selecting optical parameters from the literature is an error-prone task. Glaring examples are the absorption and scattering parameters, which are mostly determined in *ex vivo* tissue samples and may differ by an order of magnitude^36,37^. Moreover, the scattering coefficients of living skin can be much lower than those of *ex vivo* samples^38,39^.

**Figure 8:**
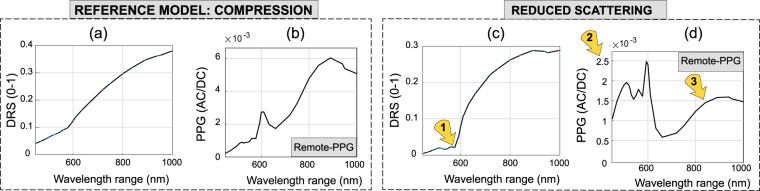
Spectral effects due to uncertainty in the epidermal scattering coefficient. (**a**,**b**) Baseline *μ*_*s*,*EPI*_ = 156.34 cm^−1^ versus (**c**,**d**) Alteration *μ*_*s*,*EPI*_ = 10.42 cm^−1^. The yellow flags point to differences in 1. DR at green wavelength range, and 2,3 PPG-amplitude magnitude and ratio between blue-green versus red-IR windows.

Although the major findings are not affected, the uncertainty in parameter settings influences the DRS and PPG spectra. The computation of the skin layer’s signatures is robust to small variations in blood concentration at the upper dermis. However, the same does not hold if the skin layers scattering or absorption coefficients vary by an order of magnitude. Figure 8 exemplifies the considerable impact on spectral simulations of an hypothetical variation in epidermal scattering by an order of magnitude (reference *μ*_*s*,*EPI*_ = 156.34 cm^−1^ versus reduced *μ*_*s*,*EPI*_ = 10.42 cm^−1^; common parameters for the epidermal layer: n = 1.33; *μ*_*a*,*EPI*_ = 15.039; g = 0.9; *d*_*EPI*_ = 0.8 mm).

The errors incurred in parameter settings do not preclude the remote PPG spectrum from being obtained. However, the errors propagate to the mixing weights of the BVVs. Consequently, the inverse estimation of the mixing weights of the BVVs is currently an ill-posed problem. Future progresses in this direction could enable the possible usefulness of the skin’s pulsating profile for functional tissue characterization.

#### Probe effects

DR measurements were contact-based and may contain probe pressure artifacts^40,41^. Concerns are rested by verifying that the DR plot for the non-compressed skin is similar to those of Bjorgan *et al*.^42^. Probe effects reduce the PPG-amplitude ratios in green over red-IR by a factor of up to 0.7 (corresponding to about 1 mm of finger compression; see Supplementary section 1 for details on the PPG-amplitude response to incremental finger pad compression in green and IR). Modeling the geometry of the OFP (implicit in TF computations) is another possible source of error which makes it inviting to perform of spectral measurements remotely as demonstrated by Corral *et al*.^43^ and Blackford *et al*.^44^. However, measuring the PPG spectra in reflectance-mode was preferable to remote multispectral measurements since the latter are noisier. Concerns to the validity of the remote spectra PPG include ballistocardiographic artifacts^17^ and specular reflections.

## Conclusion

The exploration of the VIS range in PPG-based applications is relatively recent and the underlying opto-physiology remains doubtful. Our results present a step forward in this regard by supporting the volumetric model. By taking a joint numerical and experimental approach, we linked the skin’s pulsating profile and signatures at different skin-depths with the remote PPG-amplitude spectrum. Our results support that arteriole-arterial BVVs are feasible as origin of PPG signals in visible light and IR. The depth-origin of PPG using green wavelengths are dermal BVVs while red-IR wavelengths even interact with subcutanous BVVs. The videocapillaroscopic mapping of the PPG-amplitude at the finger nail fold further suggests that the PPG signal is not associated with capillary density.

## Methods

### Participants

Sixteen subjects (ages, 27–55 years old; 2 females) participated in this investigation. The study was approved by the Internal Committee Biomedical Experiments of Philips Research and an informed consent was obtained from each subject. All experiments were carried out in accordance with the ethical standards laid down in the 1964 Declaration of Helsinki.

### Data acquisition

We explored three modalities for acquiring skin reflectance data. Camera-based remote PPG measurements were made in green versus IR bands. Because of its relevance for prospective PPG-imaging applications, the derived insights are the foundation of this study. Videocapillaroscopy offers a joint morphological and functional assessment of the upper dermis and of the associated remote PPG signal strength. DRS extends insights to the 500–940 Hz spectra, and strengthens our study by making the skin compression intervention possible.

This section details the data acquisition settings used in this investigation. We highlight that, in all data acquisition modalities, the sampling rates in the range 16–30 Hz are well above the Nyquist frequency (about 1 Hz) and enable estimating the average pulse-rate frequency using Fourier analysis in each temporal window of 10–20 sec. The average diastole and systole instants are determined with a precision error of 63 ms, which is appropriate for noise mitigation and imaging PPG.

#### Camera-based remote PPG measurements

Skin video recordings were performed using a monochrome camera (IDS Inc, Germany; model *μ*Eye, UI122xSE-M; 12 bit resolution; model, USB 2000+; sampling rate, 20 or 30 Hz). The susceptibility of remote PPG to motion artifacts was addressed in finger pad recordings by supporting the forearm on a table. Additional care was taken to eliminate specular reflections by including polarizing film in front of the light source and camera in a cross-polarization arrangement. For each video recording, skin regions of interest (sRoIs) were manually demarcated at video recordings of the finger pad and used to extract PPG signals. For each frame, the raw PPG signal is computed by averaging the time-varying intensity of the sRoI in successive frames. Each camera channel retrieves a single time series per sRoI.

#### Videocapillaroscopy

Recordings were performed at the finger nail fold of one subject from our dataset. Our system comprised a CMOS camera (coupled with a green filter; sampling rate, 20 Hz), a microscope lens (magnification factor, 40x) and a white high power LED ring light (CCS HPR2-100SW; fluorescent white; voltage, 24 VDC; see Fig. 4a). Motion artifacts were addressed by supporting the forearm on a table. Minute cardiac-related bulk motion cannot be fully suppressed, but the diffuse lighting at the imaged finger nailfold prevents artifacts in remote PPG signals and amplitude maps. Specular reflections were minimized by applying a thin layer of ultrasound gel at the nail fold and a microscope slide, which promote the translucency of the epidermis (refractive index = 1.3) and by using a LED-ring close to the finger site (distance of about 1 cm). The orientation of the LEDs (70–80 degrees w.r.t. the camera, which is frontal to the nailfold) helped to minimize specular reflections. The observation that PPG originates from non-capillary regions within videocapillaroscopic data and the derived PPG-amplitude images was confirmed in different measurement sessions and fingers.

#### DRS

Contact-based reflectance data was probed with a spectrometer (Ocean Optics, Inc.; model, USB 2000+; 12 bit resolution; software, SpectraSuite, 2008) coupled with an OFP. For each recording, we obtained parallel sets of *N* = 2048 time series at collocated, narrow and non-overlapping frequency bands, including the 475–975 nm range. The OFP (diameter, 400 *μ*m) was shielded by a cylindrical ferrule which blocked ray paths back-scatted up to 1.59 mm from its center.

The finger pressures applied with our DRS-OFP equipment at normal/reference measurement conditions at the finger pad are low and estimated to be below 10 kPa (finger compression depth, below 1 mm). In contrast, the applied pressure intensity used for eliciting the compression regime in the DRS reference-compression intervention is estimated to be within about 40 to 60 kPa (compression depth, about 2 mm). These compression estimates were based on a devoted experiment of PPG-amplitude and DR measurements under gradually increasing finger pad compression (see Supplemental Section 1).

### Processing skin reflectance data

Our experimental data is raw skin reflectance (sampled well above the pulse-rate frequency) from which we aim to ensemble PPG signals. The nuances in signal processing requirements for camera-based PPG acquisition and DRS are described below.

#### Camera-based video recordings

Raw PPG signals in video recordings were extracted by averaging pixels in used-defined sRoIs and AC/DC-normalized (i.e., divided by its slowly-varying component, obtained with low-pass-filtering with a Butterwordth filter with cuttoff frequency at ~40 bpm). The amplitude of the reflection-mode PPG signals is typically low (particularly in red wavelengths), but its signal-to-noise ratio (SNR) can be improved by adaptive bandpass filtering (ABPF). ABPF consists of filtering out the frequency components of the signals that are not multiples of the fundamental of the pulse-rate frequency.

ABPF was applied in an overlap-and-add manner (stride length, 256 samples; overlap factor, 50%) with Hanning windowing. We selected the fundamental and 6 harmonics of the pulse-rate frequency and a tolerance band of one bin around each center frequency. When available, the reference signals used for identifying the systolic peaks and instantaneous pulse-rate were finger pulse oximetry signals (acquired synchronously with video recordings). Alternatively, the reference were the PPG-signals probed at green wavelengths, as these have the best available SNR. After ABPF, the PPG cycles in streams were condensed into ensemble-averaged (EA) waveforms, separately for each camera channel and signal dimension. EA relies on Gaussian noise cancelation in the averaging process and in the signals periodicity. In practice, cycles were demarcated based on the timing of the systolic peaks (identified in reference signals), temporally registered, and, finally, averaged by using the trimmed mean operator (outlier rejection, 10%). Each super-resolved EA waveform condenses, at least, 100 consecutive cycles.

#### Processing of DRS recordings

As a preprocessing step, DRS streams require calibration for additive sensor noise. This is done by subtracting the noise floor in each wavelength, *λ*. By denoting the raw reflectance as *DR*_0_(*t*, *λ*), its calibration is expressed as

1 $$DR(t,{\rm{\lambda }})=\frac{D{R}_{0}(t,{\rm{\lambda }})-D{R}_{DARK}({\rm{\lambda }})}{D{R}_{REF}(t,{\rm{\lambda }})-D{R}_{DARK}({\rm{\lambda }})},$$

where *DR*_*DARK*_(*λ*) denotes the noise level in full darkness. Similarly, *DR*_*REF*_(*λ*) denotes the reflectance measurement of a reference standard (model WS-1, reflectivity >98% from 250–1500 nm, Ocean Optics, Inc.) placed frontally to the OFP at a distance of 2 mm. DR recordings were processed to obtain reflectance PPG-amplitude and PPG-phase spectra.

### Monte Carlo simulations of skin reflectance and remote PPG

The spectrum and depth-origin of remote PPG was simulated by the Monte Carlo method. These efforts were preceded by Huelsbusch^23^, but his assumption that the remote PPG signal comes exclusively from the capillary loops, as well as his overestimated blood concentration and scattering coefficients of the skin, resulted in the underestimation of PPG signals in red-IR. We overcame these issues using parameters from the DRS literature and/or estimated based on our experiments. We simulated the photon migration through the tissue for the diastolic and systolic states at a set of tissue characteristics, including SpO_2_ and blood concentration in the tissue, where the systolic state was obtained by an incremental increase of arterial blood over the diastolic state. Data were computed over multiple skin layers and in the 450–1000 nm range.

We used the publicly available GPU-MCML code package, which enables simulating photon propagation in a multi-layered turbid media with adjustable spatial dimensions and resolution^45^. The geometry of the scenario is specified in cylindrical coordinates with the emitter centered at the origin and normal to the tissue surface^33^. The emitter configuration approximates collimated light from an infinitely narrow beam. The input (.mci) files for MCML incorporated skin architecture parameters, absorbance and scattering coefficients, and physiological parameters such as SpO_2_, blood concentration, melanin content, etc. Separate models were implemented for reference and compressed skin.

#### Tissue Model

Figure 2 illustrates a multilayered model of normal glabrous (non hairy) skin tissue. The model consists of six homogeneous layers with different fractions of water, *C*_*w*_, blood, *Cb*, and fat, *Cf*. Table 1 lists layer thicknesses, water and blood fractions for each skin layer–numbered from 1 to 6 (deepest)–in the diastolic state. The first layer listed is the epidermis. At the palm or finger pad, its thickness is high in comparison with other skin sites^46^. The palm also features a fivefold lower density of melanocytes than at other skin areas^47^. Accordingly, melanin was not included in our model. For the remaining layers, the optical and anatomical properties of our skin geometry are similar to previous work^21,23,48^, though the average blood concentration in the dermis, *C*_*b*_, is lower. This setting conforms with recent DRS studies^42,49^ indicating that *C*_*b*_ is within 1–3%.

**Table 1:** Layer settings for normal/reference skin.

Skin layers	*n*	*d*_*l*_ (cm)	*C*_*b*_	*C*_*w*_	*v*_*d*_ (*μ*m)
1-EPI	1.33	0.08	0	0.20	0
2-CL	1.37	0.015	0.004	0.65	10
3-UP	1.40	0.008	0.02	0.65	20
4-RD	1.40	0.12	0.004	0.65	20
5-DP	1.40	0.05	0.04	0.65	40
6-SC	1.44	0.5	0.03	0.05	50

The refractive index for all internal surface interfaces increases gradually from 1.33 at the surface to 1.44 at the bottom interface. The arterio-venous ratio (*r*_*a*_:*r*_*v*_) corresponds to the diastolic state and was applied to all dermal layers in the diastolic state^50^. For the reference condition, *r*_*a*_:*r*_*v*_ was set at 50%:50%. Additional settings are as follows: the arterial oxygen saturation SpO_2_ was set at 97% and the venous oxygen saturation, SvO_2_, was set 30% lower; and the fat concentration, *C*_*f*_, was set at 40% at the subcutis^42^. The vessel diameters per dermal layer, *v*_*d*_, were estimated from the literature^51^.

Table 2 lists the adaptations made to mimic the skin compression status. In short, the dermal water and blood volume concentrations reduced, whereas pooling of blood (mostly venous) was implemented at the subcutis. The *r*_*a*_:*r*_*v*_ ratio was set at 100%:0 at the dermal layers and 75%:25% at the SC.

**Table 2:** Layer settings for compressed skin.

Skin layers	*n*	*d*_*l*_ (cm)	*C*_*b*_	*C*_*w*_	*v*_*d*_ (*μ*m)
1-EPI	1.33	0.08	0	0.05	0
2-CL	1.37	0.008	0.0012	0.15	10
3-UP	1.40	0.004	0.0024	0.15	20
4- RD & DP	1.40	0.1	0.024	0.15	20
5-SC	1.44	0.2	0.036	0.35	40

#### Absorption settings

The absorption coefficients of the skin layers were set differently for the epidermis and for the dermal layers. The epidermal absorption coefficient, *μ*_*a*,*EPI*_, was estimated as a combination of background tissue, *μ*_*a*,*base*,*EPI*_, and water:

2 $${\mu }_{{a},{EP}I}({\rm{\lambda }})={{C}}_{{w}}{\mu }_{{a},{water}}({\rm{\lambda }})+(1-{{C}}_{{w}})\,{\mu }_{{a},{base},{EPI}}({\rm{\lambda }}).$$

*μ*_*a*,*water*_ was determined from Palmer^52^ and Smith^53^. The baseline tissue absorption for the epidermis, *μ*_*a*,*base*,*EPI*_, translates the effect of connective tissue and was implemented from Jacques^37^:

3 $${\mu }_{a,base,EPI}({\rm{\lambda }})={\rm{\gamma }}\,[0.244+85.3\,\exp (-\frac{{\rm{\lambda }}-154}{66.2})]\mathrm{.}$$

The wavelength, *λ*, is specified in nm and the factor *γ* = 0.5 accounts for water losses during *ex vivo* measurements. The absorption coefficient for the dermal layers and subcutis during diastole, ${\mu }_{a}^{(d)}(l,{\rm{\lambda }})$, *l*2 … 6, were estimated as a sum of non-blood tissue absorption coefficient, ${\mu }_{a}^{(d)}(l,{\rm{\lambda }})$, and blood absorption, weighted by their respective concentrations within the layer. For convenience, the subscripts (*l*) and (*λ*) are omitted in the remainder of this section. ${\mu }_{a,nb}^{(d)}$, is set as follows:

4 $${\mu }_{a,nb}^{(d)}={C}_{f}\,{\mu }_{a,fat}+(1-{C}_{f})\,{C}_{w}\,{\mu }_{a,water}+\mathrm{(1}-{C}_{f})\,\mathrm{(1}-{C}_{w}){\mu }_{a,base}\mathrm{.}$$

For the dermal background absorption, *μ*_*a*,*base*_, the exponential dependency of Eq. 3 was taken from Salomatina *et al*.^54^:

5 $${\mu }_{a,base}={\rm{\gamma }}\frac{{C}_{w}}{{C}_{w0}}\,[0.244+16.82\,\exp (-\frac{{\rm{\lambda }}-400}{80.5})]$$

where *γ* was set as 0.5 for dermal layers (*l* = 2 … 5) and 0.25 for the subcutis (*l* = 6). The coefficient *C*_*w*0_ = 0.65 accounts for the fact that background measurements of *μ*_*a*,*base*_(*l*) are performed at about 65%. These settings are aimed at meeting the absorption coefficient measurements of the bloodless dermis and subcutis of Simpson *et al*.^55^.

We account for the fact that a fraction of the incident light is reflected in the vessel walls, meaning that the *apparent* blood volume that interacts with light is lower than the actual blood concentration at the skin. This effect is called self-shielding and is in conflict with the assumption of homogeneous mixture between bloodless skin tissue and blood^56^. A correcting factor for this effect is easily performed by setting a function, *f* [.], that translates effective dermal blood concentration (*Cb*) *apparent* (*Cb*′). *f* [.] is influenced by the product of the average vessel diameter and by the blood absorption of the layers, *μ*_*a*_*v*_*d*_. For collimated light, *f* [.] is an exponentially decaying function given by

6 $$f[{\mu }_{a}{v}_{d}]=\frac{1}{1+\mathrm{1.007(}{\mu }_{a}{v}_{d}{\mathrm{/2)}}^{1.228}}\mathrm{.}$$

Self-shielding is negligible in the red-IR range since *μ*_*a*_ is very low; i.e., *f* [*μ*_*a*_*v*_*d*_]≈1. For wavelengths at the 500–580 nm range, *f* [*μ*_*a*_*v*_*d*_] reaches about 0.7 at the LD and SC (where the vessel diameter is ~40 *μ*m) but only about 0.85 at the upper dermis, which is where most blue-green photons interact with tissue. Thus, the discrete absorbers correction has a minor influence on the accuracy of PPG simulations, although we implemented it for the sake of completeness. Accordingly, the diastolic arterial and venous blood fractions, ${f}_{a}^{(d)}$ and ${f}_{v}^{(d)}$, and the *Cb*′^(*d*)^ at pulsating layers were set as follows:

7 $${f}_{a}^{(d)}={r}_{a}\,Cb\,f[{v}_{d}\,(\mathrm{(1}-Sp{O}_{2}){\mu }_{a,Hb}+Sp{O}_{2}{\mu }_{a,Hb{O}_{2}})],$$

8 $${f}_{v}^{(d)}={r}_{v}\,Cb\,f[{v}_{d}\,(\mathrm{(1}-Sv{O}_{2}){\mu }_{a,Hb}+Sv{O}_{2}{\mu }_{a,Hb{O}_{2}})],$$

9 $$C{b^{\prime} }^{(d)}={f}_{a}^{(d)}+{f}_{v}^{(d)}\mathrm{.}$$

Using the absorbance spectra of deoxygenated and oxygenated hemoglobin, *μ*_*a*,*Hb*_ and ${\mu }_{a,Hb{O}_{2}}$, respectively, compiled by Bosschaart *et al*.^57^, the diastolic absorption coefficient of the total tissue was given by

10 $$\begin{array}{c}{\mu }_{a}^{(d)}={f}_{a}^{(d)}\,(\mathrm{(1}-Sp{O}_{2})\,{\mu }_{a,Hb}+Sp{O}_{2}{\mu }_{a,Hb{O}_{2}})+\ldots \\ {f}_{v}^{(d)}\,(\mathrm{(1}-Sv{O}_{2})\,{\mu }_{a,Hb}+Sv{O}_{2}{\mu }_{a,Hb{O}_{2}})+Cb^{\prime} \,{\mu }_{a,water}+\mathrm{(1}-Cb)\,{\mu }_{a,nb}^{(d)}\mathrm{.}\end{array}$$

Systole is modeled as fractional pulsatile increases, *p*, of arterial blood in pulsating layers. The systolic ${f}_{a}^{(s)}$ and *Cb*′^(*s*)^ are

11 $${f}_{a}^{(s)}={f}_{a}^{(d)}+p\,f[{v}_{d}\,(\mathrm{(1}-Sp{O}_{2}){\mu }_{a,Hb}+Sp{O}_{2}{\mu }_{a,Hb{O}_{2}})],$$

12 $$C{b^{\prime} }^{(s)}=Cb^{\prime} +p\,f[{v}_{d}\,(\mathrm{(1}-Sp{O}_{2}){\mu }_{a,Hb}+Sp{O}_{2}{\mu }_{a,Hb{O}_{2}})]\mathrm{.}$$

Two possible mechanisms ensure model consistency during the systolic increase of arterial blood volume. Either pulsatile changes are compensated by water displacements^50,58^ (WD) or there is layer expansion (LE) to accommodate the additional fluid; i.e., micro-modulations of layers thickness^59^. In LE, the layers thickness during systole is set in proportion to *p*:

13 $${d}^{(s)}=d\,\mathrm{(1}+C{b^{\prime} }^{(s)}\,p\mathrm{).}$$

By defining the expansion factor for each layer, *E*, as *d*/*d*^(*s*)^, the absorption coefficient during systole is

14 $$\begin{array}{c}{\mu }_{a}^{(s)}=E\,[{f}_{a}^{(s)}\,(\mathrm{(1}-Sp{O}_{2}){\mu }_{a,Hb}+Sp{O}_{2}\,{\mu }_{a,{H}_{b}{O}_{2}})+\ldots \\ {f}_{v}^{(s)}\,(\mathrm{(1}-Sv{O}_{2}){\mu }_{a,Hb}+Sv{O}_{2}\,{\mu }_{a,{H}_{b}{O}_{2}})+C{b^{\prime} }^{(s)}\,{\mu }_{a,w}+\mathrm{(1}-C{b^{\prime} }^{(s)})\,{\mu }_{a,nb}^{(d)}\mathrm{].}\end{array}$$

**Figure 9:**
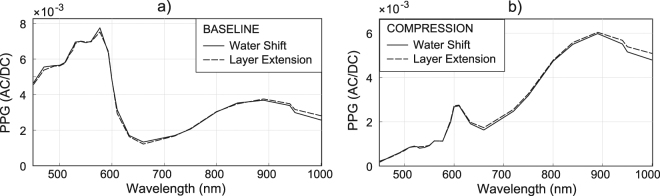
The similarity of simulation outcomes with WS and LE on (**a**) normal and (**b**) compressed skin models.

In practice, the simulations obtained under WS or LE are similar (see Fig. 9). This is unsurprising since the blood concentration at the dermis is only about 2–3% and *E* is close to unity. Accordingly, only LE was implemented.

#### Scattering settings

Skin scattering is conceptually regarded as a summation of Rayleigh and Mie scattering^60^. In spite of fundamental differences, the diffuse light setting is well approximated by assuming that scattering losses occur in the depth dimension only, thus justifying that dermal scattering is reasonably described by a one-term expression, or even set as wavelength-independent^21^. We derived the reduced scattering coefficient for the dermal tissue, ${\mu ^{\prime} }_{s}$, from the observations of Simpson and Shimada^39,55^:

15 $${\mu }_{s}\text{'}={c}_{0}(l,{\rm{\lambda }})\,{{\rm{\lambda }}}^{-b},$$

where *c*_0_(*l*,*λ*) is a calibration constant that sets the reduced scattering ${\mu ^{\prime} }_{s}$ to (EPI: 15 cm^−1^; CL, UD, RD, LD: 20 cm^−1^; SC: 10 cm^−1^)^55^. The decaying factor *b* was estimated as 0.1 below 580 nm and 0.05 otherwise. Similar to Simpson *et al*.^55^, the anisotropy factor was assumed to be 0.9 for tissue.

#### Simulating remote PPG

For each simulated wavelength, *λ* ∈ <450,1000> nm, and skin layer, *l* = 1 … 6, the relevant outputs from MCML for expressing the simulated remote PPG spectra are the fraction of photons reaching the surface per cm as a function of radial distance from the origin, *Rdr*(*r*, *λ*, *l*), and the total diffuse reflectance, *Rdt*(*λ*, *l*), expressed as fraction of total emitted photons.

Matlab was used for further processing. The diastolic-systolic diffuse reflectance outputs were applied to mimic the PPG spectra for remote and for contact-based acquisition. The remote normalized pulsatile reflectance PPG, *PPG*_*REM*_, was AC/DC normalized for pulsating layer, *l*, as normalized fractions of the total incident photons; i.e.,

16 $$PP{G}_{REM}({\rm{\lambda }},l)=\frac{Rd{T}^{d}({\rm{\lambda }},l)-Rd{T}^{s}({\rm{\lambda }},l)}{Rd{T}^{d}({\rm{\lambda }},l)}\mathrm{.}$$

where *RdT*^*s*^, and *RdT*^*d*^ denote the total diffuse reflectance during systole and diastole, respectively. Since each wavelength needs to be simulated under diastolic and systolic conditions, for a skin model with five pulsating layers at least six simulation runs were required, per wavelength. Each simulation run consisted of 10E8 to 40E8 photons and required approximately 10 min of processing time on a Linux server operating an NVIDIA GeForce GTX TITAN with compute capability 3.5 (14 SMs).

#### Optical penetration depth and depth-origin of PPG

The Matlab routines *lookmcml.m* and *getmcml.m* (publicly available at http://omlc.org/software/mc/) were used to compute the optical fluxes as a function of the skin depth from the MCML simulation output files. Care was taken to remove spurious peaks in the skin layer boundaries, thus ensuring that the flux functions are continuous along the depth axis.

For each wavelength and skin configuration, a flux function was computed for the diastolic state, *F*^(*d*)^(*z*), allowing us to express the optical penetration depth of the incident light as the skin depth, oriented along the z-axis, that is reached by $1-\frac{1}{e}$ of the incident photons. Mathematically, the PD was obtained by solving the following equation:

17 $$\frac{\sum _{z=0}^{PD}{F}^{(d)}(PD)}{\sum _{z=0}^{{T}_{s}}{F}^{(d)}(z)}=1-\frac{1}{e}\approx \mathrm{63.2 \% ,}$$

where *T*_*s*_ is the total tissue thickness. The depth-origin (DO) of the PPG signals was computed based on the flux perturbations induced during systole for each pulsatile skin layer. By weighting these according to the relative proportions of pulsatile strength in the various modeled layers, *w*_*l*_, the differential flow due to BVVs is given by

18 $${\rm{\Delta }}F(z)=\sum _{l}({F}^{(d)}(z)-{F}_{l}^{(s)}(z))\,{w}_{l}^{T},$$

where ${F}_{l}^{(s)}$ denotes the flux perturbed during systole in layer *l*. Lastly, the DO of the PPG signal was determined as the depth for which the cumulative sum of Δ*F* is $1-\frac{1}{e}$; i.e.,

19 $$\frac{\sum _{z=0}^{DO}{\rm{\Delta }}F(DO)}{\sum _{z=0}^{{T}_{s}}{\rm{\Delta }}F(z)}=1-\frac{1}{e}\mathrm{.}$$

### Indirect measurements of the remote PPG spectra by using transfer functions

Since the used OFP is shielded by a ferrule (which clips shallow photon paths) a correction is needed if the reflection-PPG spectra, *PPG*_*OFP*_, are to be used for drawing considerations to the remote setting. In this investigation, the [pseudo] remote PPG-amplitude spectrum, *PPG*_*REM*_, is estimated from *PPG*_*OFP*_ based on a transfer function such that *TF* = *PPG*_*REM*/*PPG*_*OFP*_. The numerical estimation of such TF began with simulating, in MCML, the remote diffuse reflectance (DR) in the 450−1000 nm. The uncalibrated remote DR from the skin, during systole and diastole, were obtained as functions of the source distance. Those were integrated from *Rd*_*r*_(*n*_*r*_, *λ*) as

20 $$uD{R}^{d}(i,{\rm{\lambda }})=\sum _{{n}_{r}=i}^{R=3000}R{d}_{r}^{d}({n}_{r},{\rm{\lambda }}),$$

21 $$uD{R}^{s}(i,{\rm{\lambda }})=\sum _{{n}_{r}=i}^{R=3000}R{d}_{r}^{s}({n}_{r},{\rm{\lambda }}),$$

where the index *n*_*r*_ refers to source distance. For a radial resolution of 0.0005 cm and 3000 grid points, the spanned radius ranges up to 1.5 cm. The correction factors for diastolic and systolic DR, *C*^*d*^(*λ*) and *C*^*s*^(*λ*), are obtained as

22 $${C}^{d}({\rm{\lambda }})=Rd{T}^{d}({\rm{\lambda }})/uD{R}^{d}({n}_{\varepsilon },{\rm{\lambda }}),$$

23 $${C}^{s}({\rm{\lambda }})=Rd{T}^{s}({\rm{\lambda }})/uD{R}^{s}({n}_{\varepsilon },{\rm{\lambda }}\mathrm{).}$$

with *n*_*ε*_ set to 10 to prevent numerical inaccuracies. The calibrated DR for systole and diastole becomes

24 $$D{R}_{OFP}^{(s)}({\rm{\lambda }})={C}^{(s)}({\rm{\lambda }})\,uD{R}^{(s)}({n}_{r0},{\rm{\lambda }}),$$

25 $$D{R}_{OFP}^{(d)}({\rm{\lambda }})={C}^{(d)}({\rm{\lambda }})\,uD{R}^{(d)}({n}_{r0},{\rm{\lambda }}\mathrm{).}$$

with *n*_*r*0_ = 278. Finally, the reflectance PPG signal the OFP and layer *l* was obtained as

26 $$PP{G}_{OFP}(l,{\rm{\lambda }})=\frac{D{R}_{OFP}^{(d)}({\rm{\lambda }})-D{R}_{OFP}^{(s)}({\rm{\lambda }})}{D{R}_{OFP}^{(d)}({\rm{\lambda }})}\mathrm{.}$$

The TF from reflectance to [pseudo] remote-PPG was finally given by

27 $$TF({n}_{r0},{\rm{\lambda }})=\frac{\sum _{l}PP{G}_{REM}(l,{\rm{\lambda }}){w}_{l}}{\sum _{l}PP{G}_{OFP}(l,{n}_{r0},{\rm{\lambda }}){w}_{l}}\mathrm{.}$$

**Figure 10:**
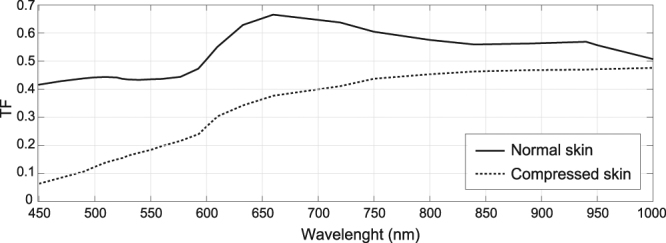
TF functions for converting reflection-mode to remote PPG spectra. These describe the “distortion” effect introduced by the OFP’s ferrule on the PPG spectrum and evidence the boosting of, mostly, PPG-amplitudes <600 nm.

The compression curves for normal and compressed skin are shown in Fig. 10. Both indicate that the OFP configuration boosts the PPG-amplitude, particularly in blue-green wavelengths.

#### Simulation precision

The average simulation errors for the remote PPG spectra–expressed as standard deviations over the means–is 4.7% for the 475–1000 nm range. This error estimate was based on four repeated runs of the compressed skin model.

## Electronic supplementary material

Supplementary File
