# Supplementary material for: New insights into the origin of remote PPG signals in visible light and infrared
Source: Sci Rep. 2018 May 31;8:8501. doi: 10.1038/s41598-018-26068-2 (PMC5981460; doi:10.1038/s41598-018-26068-2)
Supplement: Supplementary file 1 — Supplementary File [file 41598_2018_26068_MOESM1_ESM.pdf]

New insights into the origin of remote PPG signals in visible light and infrared

Andreia V. Moço[1,*], **Sander Stuijk**[1], **and Gerard de Haan**[1,2]

[1]Department of Electrical Engineering, Eindhoven University of Technology, 5612AZ Eindhoven, the Netherlands
[2]Philips Innovation Group, Philips Research, High Tech Campus 36, 5656AE Eindhoven, the Netherlands
[*]a.moco@tue.nl

Supplementary section 1: PPG-amplitude response to increasing skin compression

Experimental setup This subsection describes an experiment to illustrate the effect of gradually increasing compression on the perfusion status of the skin tissue. We measured the finger pad under increasing compression and assessed the normalized amplitude of remote PPG signals acquired in green and IR, as well as skin reflectance.

A subject was asked to have his right pointing finger tilted upwards and covered by a light glass plate, while refraining from voluntary motions for 10 consecutive compression steps of 2 min (see Supplementary Fig. 1). An initial step was done without contact to the glass plate. Subsequently, the pressure against a glass plate were gradually incremented in a contact area ranging from 0.5×1 cm^2 to 1×1.5 cm^2 (compression was increased in steps of 2 mm, each corresponding to about ~ 5 kPa). Illumination was provided by 2 fluorescent lamps (Philips EnergyLight, reference HF3319). Two independent monochrome cameras were used, each capturing the finger pad at a rate of 15 frames per second and with 8 bits depth (camera 1, model μEye of IDS Inc., Germany, coupled with a bandpass filter at 559/34 nm; camera 2, model Marlin F046C, Allied of Vision Technologies GmbH, Germany), coupled with a high pass filter with cutoff frequency at 840 nm).

Recordings were processed separately for each compression stage. The instantaneous pulse-rate was estimated by using a finger oximeter, synchronized with the camera. PPG signals were AC/DC-normalized and digitally band-pass filtered around the fundamental pulse-rate frequency. In experiments, the finger was at the level of the heart.

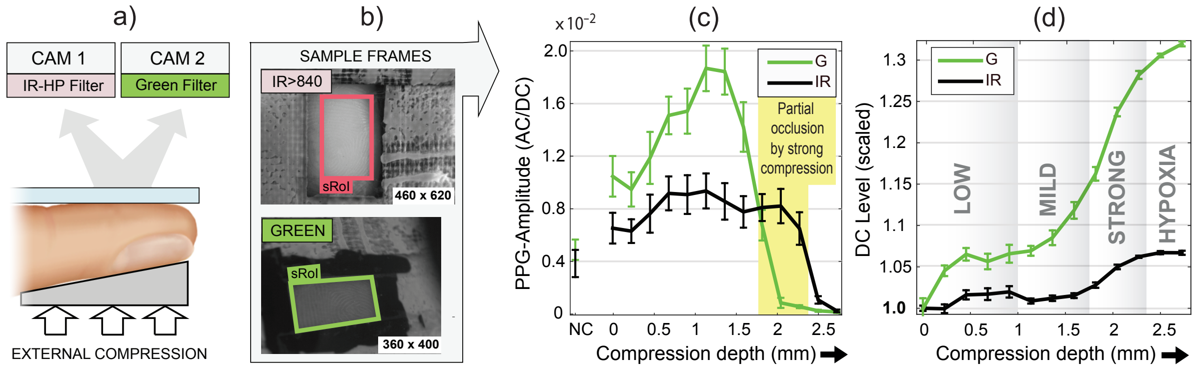

Supplementary Figure 1. (a) Setup for simultaneous acquisition of PPG signals of the compressed finger pad (against a glass plate), with (b) two monochrome cameras coupled by R and green filters; (c, d) PPG-amplitude and DC Level (i.e., skin reflectance), as function of compression depth, featuring the states of low-mild-strong compression and tissue hypoxia. At strong compression, the PPG-amplitude in green falls abruptly, while PPG-IR remains stable.

Results and discussion Supplementary Figure 1 summarized our results for PPG-amplitude and relative skin reflectance for increasing levels of skin compression (measured site, finger pad). The PPG-amplitude curve suggests three regimes which we qualitatively classify as low, mild and strong compression. During low-mild compression, increasing compression results in the rising part of the normalized PPG-amplitude curves in green wavelengths. The DR enables us to differentiate low and mild states by the timing at which the green and IR DR curves begin to increase exponentially (see Supplementary Fig. 1b. This behaviour is understood by recalling that the capillary bed of tissue gradually becomes devoid of the non-pulsatile venous blood, while arterioles remain pulsatile. This leads to a decrease in the optical density of the upper dermis and a resulting increase in light penetration depth of all wavelengths. As the reflected light becomes modulated by deeper vessels and the relative fraction of arterial pulsations (with respect to the total blood volume) in the skin increases, the PPG signals become

stronger. Compression gains are most dramatic in green because IR wavelengths were already able to reach deeper vessels, thus being less sensitive to the initial stages of applied compression.

The end of the low-mild regime is seen as an abrupt PPG-amplitude decrease in green and as an exponential increase in DC-reflectance. For strong compression, the skin is partially occluded in the sense that only blood volume variations sources (BVVSs) at deeper layers remain pulsatile. For compression above systolic blood pressure, the digital artery is occluded and the tissue becomes ischemic. This behavior were verified in two additional subjects, although the critical compression depths demarcating each state differ due to differences in total finger thickness.

Supplementary section 2: Variations between multi-site PPG-amplitude spectra

We explored the skin-site influence on PPG-amplitude spectral measurements by performing three repeated PPG-amplitude measurements at four skin locations: the finger pad and nail fold (non-glabrous skin), and forehead and cheek (glabrous skin). Measurements were done in the same test session and subject (female; skin type 3 at the Fitzpatrick scale; age, 32 yrs) under stable room temperature. The reflection-mode PPG spectra was averaged, separately for each skin site, and scaled by unity at the 660-700 nm range. Supplementary Fig. 2 contrasts the resulting face and finger measurements and suggests that IRoR is more insensitive to skin locations than GoR.

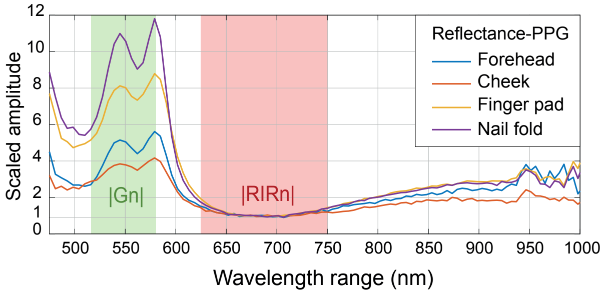

Supplementary Figure 2. Differences between PPG-amplitude spectra acquired at the forehead and finger. Amplitudes are stronger at the finger that at facial locations, and that GoR and IRoR are also skin-site dependent.

Supplementary Table 1 summarizes the obtained GoR and IRoR for the 4 skin sites and confirms that the inter-site variations in GoR go up to a factor of 2.71. For the same conditions, IRoI is more stable and varies only up to 1.41.

Supplementary Table 1. Amplitude ratios for facial skin sites at the face and finger.

PPG-amplitude ratios	forehead	cheek	finger pad	nail fold
$\lvert PPG,IR\rvert/\lvert PPG,R\rvert$	4.99	3.80	7.97	10.32
$\lvert PPG,G\rvert/\lvert PPG,R\rvert$	2.16	1.77	2.51	2.41

Supplementary Fig. 3 contrasts the EA waveforms for green (520–580 nm) and red-IR (625–750 nm) ranges, which also suggest waveform differences between skin sites.

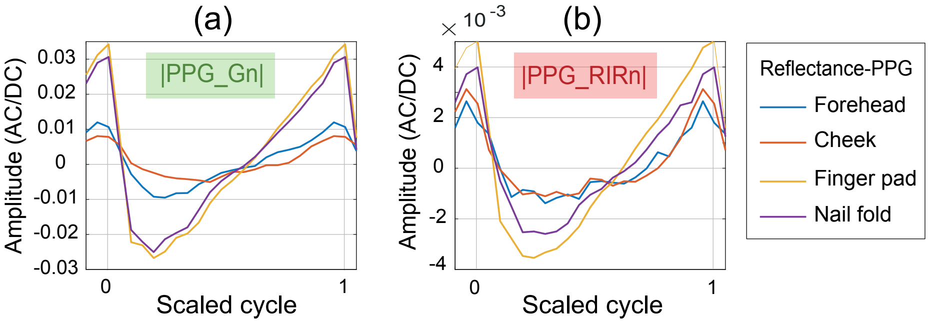

Supplementary Figure 3. Multi-site waveforms of normalized green ($|Gn|$) and red-IR ($|RIRn|$) in reflection-mode PPG.
